# rs779805 Von Hippel-Lindau Gene Polymorphism Induced/Related Polycythemia Entity, Clinical Features, Cancer Association, and Familiar Characteristics

**DOI:** 10.3389/pore.2021.1609987

**Published:** 2021-11-26

**Authors:** Gyula Remenyi, Zsuzsanna Bereczky, Réka Gindele, Aniko Ujfalusi, Arpad Illes, Miklos Udvardy

**Affiliations:** ^1^ Division of Hematology, Department of Internal Medicine, Faculty of Medicine, University of Debrecen, Debrecen, Hungary; ^2^ Division of Clinical Laboratory Science, Department of Laboratory Medicine, Faculty of Medicine, University of Debrecen, Debrecen, Hungary; ^3^ Division of Clinical Genetics, Department of Laboratory Medicine, Faculty of Medicine, University of Debrecen, Debrecen, Hungary

**Keywords:** single nucleotide polymorphism, secondary erythrocytosis, von Hippel-Lindau gene, rs779805 polymorphism, phlebotomy

## Abstract

Increased red blood cell count may result from primary erythrocytosis (polycythemia vera), but it is often due to secondary causes with increased erythropoietin levels. Secondary erythrocytosis may also be congenital due to different gene mutations of hemoglobin, hemoglobin stabilization proteins, EPO receptors, or oxygen sensing pathways. Von Hippel- Lindau gene mutation causes altered tissue oxygen sensation in VHL disease, usually with normal hemoglobin. Germline *VHL* mutations associate with classical VHL disease and represent genetic susceptibility for pheochromocytoma. *VHL* polymorphisms are mostly considered an innocent phenomenon. Still, some data indicate that these polymorphisms are not always harmless and can occur with prostate, renal, and colon cancer or even with isolated erythrocytosis. Seventy-eight patients referred to our department with elevated hemoglobin were screened for *VHL* mutations. There were no classical somatic *VHL* mutations. However, we found heterozygous (GA) or homozygous (AA) rs779805 *VHL* c.-195G>A polymorphism accompanied by erythrocytosis. These patients are *Jak-2* negative, with normal or elevated EPO levels, sometimes with family accumulations and often phlebotomy needs, and in some cases with malignancies in the family. No other cause of erythrocytosis was found. We use phlebotomy regularly, and for those with cardiovascular risk factors, we recommend aspirin.

## Introduction

In most cases, polycythemia vera (PV) diagnosis can easily be made based on WHO 2016 criteria, but the background to “simple” erythrocytosis cases is often unclear. Erythrocytosis can be primary or secondary. The leading cause of primary acquired erythrocytosis is PV with the *Jak2 V617F* driver mutation. The sporadic primary hereditary erythrocytosis is caused mainly by the *erythropoietin receptor* gene (*EpoR*) mutation. Increased erythropoietin (EPO) level can lead to secondary erythrocytosis from different disorders, i.e., chronic lung and heart disease, chronic increase in carbon monoxide due to smoking, sleep apnea, renal cysts and tumors, liver diseases, high-altitude living, and EPO-secreting tumors (cerebellar hemangioblastoma, renal cell carcinoma, hepatocellular carcinoma, or uterine leiomyoma). When these common causes of secondary erythrocytosis are excluded, an inherited cause involving hemoglobin or erythrocyte regulatory mechanisms may be suspected. Several genes of hemoglobin, hemoglobin-stabilization proteins, EPO receptor, or oxygen-sensing pathway enzymes may be affected. These are the *von Hippel-Lindau gene* (*VHL*) (the first described), *EGLN1/PHD2*, *EPAS1/HIF2A*, *EPO*, and *EPOR* genes (1,2). Inherited erythrocytosis comes with isolated red blood cell increase and normal spleen size, and it is generally believed that the risk of clonal evolution does not accompany it. A small subset of cases is associated with pheochromocytoma and paraganglioma formation. Although the incidence of thrombotic events may be higher, the use of regular phlebotomy and aspirin has not been proven (3). The *VHL* encodes a protein complex component involved in the ubiquitination and degradation of a hypoxia-inducible factor (HIF). This transcription factor plays a central role in the regulation of gene expression by oxygen. Von Hippel-Lindau (VHL) disease is a complex disorder in which increased oxygen demand of tissues results in a complex set of neoplastic disorders: i.e., clear cell renal carcinoma, pheochromocytoma, hemangioblastomas, pheochromocytoma, or pancreas cyst appearing well before the age of forty, and usually without erythrocytosis. The background of this disease is genetic, caused by a series of mutations in the *VHL* gene, which is positioned on the short arm of chromosome 3 (3p25-26) (4,5). There are over 1500 germline mutations and somatic mutations found or identified in VHL disease. Some rare cases of mutated *VHL* might induce isolated polycythemia; interestingly, the other tumors are mostly missing in these cases, so it is recommended to check the *VHL* in polycythemia if etiology is not otherwise explained (6–10). Much less is known about *VHL* single nucleotide polymorphisms (SNP). They are considered usually innocent, without any clinical significance. However, *VHL* SNPs are not always innocent. At least two known SNPs are not insignificant on clinical grounds. One example is the rs779805 c.-195G>A polymorphism (genomic location: GRCh38.p13 chr 3, HGVS: NC_000003.12:g.10141653G>A). According to the ClinVar database, this variant is clinically benign. No studies concerning functional characterization have been found in the literature so far. However, this SNP is frequently associated with clear renal cell cancer in Taiwan and Mainland China, although much less in the Caucasian population (11). Somewhat more prostate and colon cancers have been found in this polymorphism as well. *VHL* rs779805 polymorphism is not more frequent in colon cancer than others (12). The “G” allele frequency of rs779805 was found to be 0.457 according to the results of the 1000 Genome project in a huge sample (*n* = 5008 sample size). However, in the subgroup analysis of *n* = 1000 European samples, it was even less, at 0.297. The “G” allele is also the minor allele in the Asian population, similar to the European data. Interestingly, the “G” allele is the major allele in the African population (allele frequency of 0.844 (https://www.ncbi.nlm.nih.gov/variation/tools/1000genomes/). *VHL* polymorphism rs1642742 has a similar disorder association and geographic distribution. However, no blood count abnormalities were mentioned in these Chinese reports (13).

### Patients and Methods

Patients were referred to us on suspicion of PV. In addition to a detailed history and blood counts, we measured EPO levels, and patients’ blood was tested for *Jak2*, *CALR*, and *MPL* gene mutations. Between 2017 and 2021, the possibility of congenital erythrocytosis arose in 84 cases, so we performed a *VHL* mutation test as well, including rs779805. Other detailed studies, i.e., sequencing of genes of the oxygen-sensing pathway or NGS panel for hereditary erythrocytosis as recommended by McMullin (14), are not possible in our clinic. We eagerly screened the *VHL* in young patients or evidence of a familiar polycythemia pattern or more cancer. We screened 78 probands or probands’ relatives with erythrocytosis for *VHL* rs779805. DNA samples of 51 healthy individuals were also tested for rs779805 SNP. In the patients’ DNA sample, the coding regions of the *VHL* and the surrounding intron regions were amplified by PCR reactions, followed by direct fluorescent sequencing. For detecting mutations in the 5′-upstream region (including c.195G>A, the site of rs779805) and in the coding region of the *VHL*, DNA was isolated from whole blood leukocytes anticoagulated with sodium citrate or K3-EDTA by QIAamp DNA Blood Mini kit (QIAGEN GmbH, Hilden, Germany). After PCR amplification, direct fluorescent DNA sequencing was performed on an ABI3130 Genetic Analyzer using the same primers as were used for PCR reactions. (Oligonucleotide sequences of primers for PCR reactions are available upon request.) Sequencing Analysis 5.4 software (Thermo Fisher Scientific, Carlsbad, CA, United States) was used for sequence analysis and compared to NCBI Reference Sequences (Gene Bank accession No.: AF010238.1). Multiplex ligation-dependent probe amplification (MLPA) was also performed using SALSA MLPA KIT P016 VHL (MRC-Holland, Amsterdam, Netherlands) to exclude the presence of larger gene segment deletions not detectable by Sanger sequencing. The MLPA products were analyzed by GeneMapper Software 4.1 (Thermo Fisher Scientific). No functional characterization has been performed. Allele frequency of rs779805 was also determined in 51 healthy blood donors who did not suffer from any hematological diseases or malignancy. Variables are reported as counts and percentages. Statistical power calculation was performed with differences in the G allele ratio in the probands with erythrocytosis and probands without erythrocytosis group. Power is 89.4%, with 78 cases. Nominal variables were compared between groups using the Chi-squared or Fisher’s exact test, as appropriate. The *p*-value threshold for significance was 0.05. All statistical analyses were performed using the Statistical Package for the Social Sciences (IBM SPSS Statistics v26.0.0), United States. Power calculation was performed using GPower 3.1.9.2 software.

## Results

We have not detected any known disease-causing *VHL* mutation. However, we found, even to our surprise, among patients referred with erythrocytosis, 61 patients or probands with the same *VHL* SNP, namely the intron (c.-195G>A) before the 1st exon: i.e., rs779805 G>A, with GA or AA genotype, similar to what had been described in a Chinese population (11,13) without blood count abnormalities (but associations with solid tumors) (Tables 1–3) No other mutation or SNP has been found. We had 38 patients with idiopathic erythrocytosis, 20 of whom required regular phlebotomy. We screened some of the patients’ relatives. Out of 16 relatives, 5 had erythrocytosis as well. We used the *VHL* mutation results of 51 control cases from the Department of Laboratory Medicine (Table 1). Samples from 78 patients/relatives and 51 healthy controls were examined. Allele frequency was in Hardy-Weinberg equilibrium for both the control and patient group. The G and A allele frequency values found in our patient’s group (G allele 0.46; A allele 0.54) did not differ significantly from the values calculated for the control group (G allele 0.33; A allele 0.67), *p* = 0.063. The ratio of the minor G allele carriers (i.e., GG homozygotes and GA heterozygotes combined) was significantly higher in the patient’s group (66%) as compared to the control group (51%) (*p* = 0.026). However, when erythrocytosis and non-erythrocytosis cases were compared, the AA genotype was dominant: AA 0.37 vs. 0.15 (*p* = 0.038, power: 89.4%). In idiopathic erythrocytosis, the incidence of GG genotype was lowest, usually with a healthy family member. Only 3 out of 17 (18%) have erythrocytosis (Table 5): GG: 0.03, GA: 0.55, AA: 0.37. If there was no erythrocytosis in the proband group, these were 0.35, 0.50, and 0.06, respectively (*p* = 0.0017, power 100%). Of the 41 GA genotype patients, 21 had erythrocytosis (Table 3). Erythrocytosis and erythrocytosis requiring phlebotomy were more common in patients with *VHL* rs779805 GA or AA genotypes than the GG genotype. Interestingly, we also found one patient with polycythemia vera (PV) and one with Myelofibrosis (MF), with low EPO levels with GA genotype, and 3 PV patients with normal EPO levels with AA genotype. The Hgb values of each genotype group did not differ significantly. (Tables 3–6). We identified three families where the same SNP (2 AA and 1 GA genotype) was detectable in family members. In these families, there was an accumulation of erythrocytosis and tumors. Interestingly, either erythrocytosis or tumor occurred, but not both. We have found additional cancers in 14 families: clear renal cell cancer (1), colon cancer (5), melanoma (1), a nodular sclerosis Hodgkin lymphoma, (might or might not be coincidental), a single case of non-Hodgkin lymphoma, bony tumors, gynecological cancers, and hemangioma in first degree relatives. Some patients (during family exploration) had hemoglobin levels over 190 g/L under the age of twenty. The case of patient #10 with an AA genotype is exciting. He was diagnosed with erythrocytosis at the age of 4 and has been regularly phlebotomized ever since (more than 500 times). He has even had six trephine biopsies with indifferent results (Table 4). Upon investigating the whole *VHL*, we did not find any other genetic variation in our patients. For comparison, we examined *VHL* polymorphism in 51 healthy volunteers. Hemoglobin level at the upper limit of normal (between 150 and 160 g/L) was observed in 12 healthy individuals, and among them, only GA and AA genotypes were found (*n* = 7 GA, *n* = 5 AA).

**TABLE 1:** Genotypic distribution of samples of wild type (G/G) and heterozygous (G/A) and homozygous (AA) *VHL* rs779805 SNP. Probands were examined for erythrocytosis. *VHL* mutation analysis was performed on 16 of the available relatives. A total of 51 healthy control samples were used.

*n* = 129	GG	GA	AA
Probands (*n* = 62)	16 (26%)	33 (53%)	13 (21%)
Relatives (*n* = 16)	1 (6%)	8 (50%)	7 (44%)
Healthy control (*n* = 51)	7 (14%)	19 (37%)	25 (49%)

**TABLE 2:** Allele frequencies in 78 cases of probands/relatives with or without erythrocytosis. The AA genotype was more common in the EC group, while the GG genotype was hardly present.

	Erythrocytosis group	Non-erythrocytosis group
GG genotype	3 (8%)	14 (35%)
GA genotype	21 (55%)	20 (50%)
AA genotype	14 (37%)	6 (15%)
	*n* = 38	*n* = 40

**TABLE 3:** Patient characteristics with erythrocytosis and GA genotype of *VHL* rs779805 SNP. Out of 41 patients, 21 (51%) have erythrocytosis. Patients are all *Jak-2 V617F* unmutated and *Jak-2 exon 12*, *calreticulin*, and *MPL* unmutated with normal or low EPO level (norm. range 4.3–29.0 IU/L). Twelve have erythrocytosis with regular phlebotomy. The most common malignancies in the family are colon cancer, Hodgkin’s lymphoma, and myelodysplastic syndrome.

	Gender	Age	Age of onset	EPO level (IU/L)	Hgb (g/L)	Htc	RBC (T/L)	Clinical picture
1	M	14	8		144	0.44	5.3	his mother is AA genotype with renal cc.
2	M	56	51		188	0.54	5.79	psoriasis, MTX th.
3	M	47	42	4	199	0.58	6.11	
4	M	68	63	7.6	184	0.53	5.55	HT, Diabetes
5	M	46	36	6.7	183	0.54	6.32	COPD, smoker
6	M	61	60	32.3	172	0.55	5.85	COPD, deceased
7	M	65	59	7.7	177	0.52	5.33	Hepatopathy
8	M	21	21	4.7	173	0.52	5.31	Cong. urinary tract development disorder
9	M	27	22	14.1	175	0.50	5.25	Phlt since 2014, his sister is GA genotype, with Hodgkin lymphoma
10	M	27	18	5.9	164	0.47	5.28	Phlt, alopecia areata, his mother is AA genotype. His father is GA genotype, EC, COPD
11	F	67	50		164	0.50	5.23	Phlt since 2007, ICD
12	M	39	28	7.6	193	0.56	6.33	Phlt since 2008
13	M	17	10		165	0.50	6.14	Phlt, his mother is an AA genotype with renal cc.
14	M	39	38	7.4	198	0.60	6.3	Phlt
15	M	57	56	9.7	203	0.60	6.48	Phlt since 2018
16	M	40	30	14.6	176	0.49	5.79	Phlt since 2009
17	M	57	53	9.19	182	0.56	5.48	Phlt since 2015, HT, diabetes
18	F	53	50	3.0	187	0.56	5.76	Phlt since 2016, diabetes, hypothyroidism
19	M	78	73	8.5	176	0.53	5.74	Phlt since 2014, HT
20	M	52	49	15.7	190	0.58	6.04	Phlt since 2014, smoker
21	M	64	54	11.2	181	0.53	5.9	Smoker, stroke

MTX: methotrexate, th: therapy, EC: erythrocytosis, HT: hypertension, Phlt: phlebotomy, COPD: chronic obstructive pulmonary disease, ICD: ischemic heart disease

**TABLE 4:** Patient characteristics with AA genotype of *VHL* rs779805 G>A SNP with erythrocytosis. Fourteen patients out of 20 have erythrocytosis (70%). Patients are *Jak-2 V617F*, *Jak-2 exon 12*, *calreticulin*, and *MPL* unmutated with normal or low EPO level (norm. range 4.3–29.0 IU/L). Patient #10 has had phlebotomy since he was 4.

	Gender	Age	Age of onset	EPO level (IU/L)	Hgb (g/L)	Htc	RBC (T/L)	Clinical picture
1	F	38	29	9.6	165	0.49	6.0	Renal cc., her father is AA genotype, EC, renal cc., her sons EC, AA genotype
2	M	62	44	10.2	197	0.58	6.16	EC, his daughter EC, AA genotype
3	M	60	55	23.2	185	0.54	5.88	
4	M	44	42	13.2	173	0.52	5.82	Splenomegaly, smoker
5	M	51	44	5.2	174	0.52	6.06	
6	M	24	15	4.8	183	0.51	6.19	
7	M	64	62	19.2	188	0.55	6.48	Prostate cc., HT
8	M	39	45	6.2	176	0.5	5.85	EC since 2012, Phlt
9	M	38	35	1.9	182	0.54	6.04	Phlt.
***10***	***M***	***19***	***4***	***14.5***	***205***	***0.54***	***6.4***	***Phlt since he was 4***
11	M	46	43	1.1	195	0.59	5.8	Phlt, smoker
12	M	23	23		170	0.48	5.29	Phlt since 2019
13	M	56	55	8.4	173	0.52	5.66	Phlt since 2018
14	M	49	29	5.7	182	0.55	6.31	Phlt for 20 years

EC: erythrocytosis, HT: hypertension, Phlt: phlebotomy

**TABLE 5:** Patient characteristics with homozygous GG genotype of *VHL* rs779805 G/A SNP with erythrocytosis. Only three patients have elevated Hgb levels repeated times out of 17 (18%).

	Gender	Age	Age of onset	EPO level (IU/L)	Hgb (g/L)	Htc	RBC (T/L)	Clinical picture
1	M	33	31	6.5	179	0.52	6.32	
2	M	36	30		181	0.54	6.26	bodybuilder
3	M	43	40	7	203	0.59	6.28	Phlt since 2017

Phlt, phlebotomy.

**TABLE 6:** Phenotypic differences with wild type (G/G) and heterozygous (G/A) and homozygous (AA) *VHL* rs779805 SNP. Erythrocytosis or erythrocytosis requiring phlebotomy were more common in patients with GA or AA genotype of *VHL* rs779805 SNP.

	Normal Htc	Erythrocytosis	Erythrocytosis with phlebotomy
G/G genotype (17 pts)	14 (82%)	2 (12%)	1 (6%)
G/A genotype (41 pts)	20 (48%)	9 (21%)	12 (29%)
A/A genotype (20 pts)	6 (30%)	7 (35%)	7 (35%)

## Discussion

Rare inherited gene disorders may cause secondary erythrocytosis. These include the genes of proteins involved in oxygen-sensing pathways, such as *VHL* genes. In addition to the many mutations that cause VHL disease, many SNPs have no clinical significance. It has been shown that in real life, not all polymorphisms are benign. Some may come with malignancies (colon, renal, prostate), some with erythrocytosis. Only a few publications implicate *VHL* SNPs in inherited erythrocytosis. *VHL* mutations can cause isolated erythrocytosis. Two homozygous mutations of the *VHL*, Chuvash (*VHL*; p.R200W) and Croatian (*VHL*; p.H191D) polycythemias, both in exon 3, have been reported to cause polycythemia but not a tumor (15,16). Other *VHL* missense mutations lead to pulmonary hypertension and polycythemia (8). The polymorphism rs779805 has been firstly described in patients with Chuvash polycythemia (17) in a context of a “core haplotype” (also conserved in a patient with von Hippel-Lindau disease) (18). This polymorphism has been linked to renal cell cancer in many papers (11,19–22). GA or GG genotype compared to AA is a risk factor for renal cancer. However, the hypothesis is still discussed, notably in other types of tumors, i.e., decreased risk of prostate cancer (12,13), especially among non-smoking, non-drinking patients without of history of prostate cancer (13). Notably, its link with methylation is discussed: the polymorphism “G” is located in the promoter of the *VHL*. This gene region is rich in CpG dinucleotides, a so-called CpG island with a sequence “CAA-CG-GCC” that can be targeted by methylation (which may silence the gene expression) (22).

Here we collected data from patients referred with elevated hemoglobin of unknown origin. We could find a special *VHL* polymorphism (rs779805 G>A) without any other known classical *VHL* mutation in these patients. These patients have typical characteristics: AA or GA genotypes, high normal or above hemoglobin values (all were over 165 g/L), normal leukocyte and platelet counts, no splenomegaly, no canonical MPN mutations, i.e., *Jak-2, calreticulin, MPL*, and higher Hgb levels in GA and AA genotype groups compared to GG. Aquagenic pruritus is usually absent. Two cases had very mild, atypical itching. We collected data for 4 years, and no MPN transformation occurred, although the follow-up time was relatively short. Bone marrow examinations were performed in some cases, with normal description and analysis results. We used cautious phlebotomies, targeting <0.52 hematocrit (Htc) levels. This is also recommended by the British Society for Hematology (23). This target Htc is higher than what is suggested in PV, but in most cases, it is a non-compliant case and requires less frequent phlebotomy, which improves compliance. We also recommend 100 mg aspirin daily. All of these patients were advised smoking cessation. We performed an ultrasound and simple cancer screening every year, which we do recommend further on.

## Conclusion

We recommend *VHL* gene sequencing in young patients with otherwise unexplained (isolated) polycythemia, especially if there is any sign of familiarity or unusual precipitation of cancer in the family. We extend our family screening more profoundly, gain data on this polymorphism in more young, healthy volunteers, and screen PV patients. We plan careful hematologic and oncologic follow-up of our patients and first-degree relatives.

## Data Availability

The original contributions presented in the study are included in the article/Supplementary Material, further inquiries can be directed to the corresponding author.
